# Feasibility study of portable technology for weight loss and HbA1c control in type 2 diabetes

**DOI:** 10.1186/s12911-016-0331-2

**Published:** 2016-07-15

**Authors:** Claire L. Bentley, Olubukola Otesile, Ruth Bacigalupo, Jackie Elliott, Hayley Noble, Mark S. Hawley, Elizabeth A. Williams, Peter Cudd

**Affiliations:** School of Health and Related Research, Sheffield, Regent Court, The University of Sheffield, 30 Regent Street, Sheffield, S1 4DA UK; Safer Care, Tamworth House, 185 Shirebrook Road, Sheffield, S8 9RF UK; Department of Oncology and Metabolism, The University of Sheffield, Beech Hill Road, Sheffield, S10 2RX UK; Department of Academic Rheumatology, King’s College London, Denmark Hill, London, SE5 9RS UK

**Keywords:** Type 2 diabetes, Mobile health, Obesity, Weight, HbA1c, Feasibility

## Abstract

**Background:**

The study investigated the feasibility of conducting a future Randomised Controlled Trial (RCT) of a mobile health (mHealth) intervention for weight loss and HbA1c reduction in Type 2 Diabetes Mellitus (T2DM).

**Methods:**

The intervention was a small wearable mHealth device used over 12 weeks by overweight people with T2DM with the intent to lose weight and reduce their HbA1c level. A 4 week maintenance period using the device followed. The device records physical activity level and information about food consumption, and provides motivational feedback based on energy balance. Twenty-seven participants were randomised to receive no intervention; intervention alone; or intervention plus weekly motivational support. All participants received advice on diet and exercise at the start of the study. Weight and HbA1c levels were recorded at baseline and weeks 6, 12, and 16. Qualitative interviews were conducted with participants who received the intervention to explore their experiences of using the device and involvement in the study including the training received.

**Results:**

Overall the device was perceived to be well-liked, acceptable, motivational and easy to use by participants. Some logistical changes were required during the feasibility study, including shortening of the study duration and relaxation of participant inclusion criteria. Descriptive statistics of weight and HbA1c data showed promising trends of weight loss and HbA1c reduction in both intervention groups, although this should be interpreted with caution.

**Conclusions:**

A number of methodological recommendations for a future RCT emerged from the current feasibility study. The mHealth device was acceptable and promising for helping individuals with T2DM to reduce their HbA1c and lose weight. Devices with similar features should be tested further in larger studies which follow these methodological recommendations.

**Electronic supplementary material:**

The online version of this article (doi:10.1186/s12911-016-0331-2) contains supplementary material, which is available to authorized users.

## Background

### Overview

Obesity is the leading risk factor for Type 2 Diabetes Mellitus (T2DM) [1]. Around 62 % of the population in England and Wales are overweight or obese [2], and in the United Kingdom (UK) the overall prevalence of diabetes is around 4.5 % (with around 90 % of these expected to have T2DM) [1, 3]. The costs to the National Health Service (NHS) are significant, with around £10 billion per year spent on diabetes [1]. High prevalence and costs of T2DM are reflected on a global scale [4].

Weight loss is an important goal in the treatment of overweight and obese T2DM patients [5]. Intentional weight loss in T2DM can lead to reduced clinical symptoms, reduced use of medications, and reduced mortality risk [6]. National guidelines for T2DM treatment emphasise the integration of optimal dietary advice and increased physical activity within a multi-component diabetes management plan [3]. This multi-component approach can help individuals with T2DM to achieve HbA1c, blood pressure and lipid profiles in or near to the normal range [3].

Recently the use of mobile health (mHealth, e.g. apps on mobile phones, pedometers) for self-management of T2DM has received attention as a potentially cost-effective strategy for weight reduction and improved glycaemic control [7–11]. A recent systematic review [12] concluded that mHealth-based interventions can lead to short-term (and potentially medium-term) clinically significant weight loss for some overweight and obese adults. Portable technology enables interventions to be employed by individuals whilst going about their daily lives [13], and facilitates self-monitoring, which is acknowledged as a key component of weight loss intervention programmes. In addition interactive technology can be designed to assist people in modifying their attitudes and behaviours [14]. However, Randomised Controlled Trials (RCTs) which aim to assess the effectiveness of mHealth for weight loss typically have significant methodological weaknesses including a lack of blinding, and often, too little information on randomisation, measures to address attrition, and delivery of participant education [12].

### Study context

The current study aimed to initiate the process of evaluating a specific mHealth intervention, in order to develop more robust evidence on whether this intervention could be used to help people with T2DM lose weight and control their HbA1c levels.

The aforementioned systematic review [12] identified a promising CE (European Conformity) marked mobile digital technology for overweight/obese T2DM patients, the AiperMotion 440 [15]. Its primary impact on a user is assumed to be adding greater motivation overtly and through empowerment to self-monitor. A 2011 study (based in Germany) reported that after 6 months of using the device the intervention group of overweight/obese T2DM patients had a greater mean weight loss and greater reduction in HbA1c compared to the control group [15]. In addition, diabetic medication was discontinued or reduced in 81 % of intervention participants (compared to 0 % in the control group). These promising results require verification because of: the researchers’ close working with the device manufacturers; unknown details about the participants and operational details of the trial; potential exaggeration because of the small number of participants (*n* = 68); personal financial cost to participants for taking part; and a lack of UK-based data (which was deemed important by clinical advisors to the current project). The value of the authors’ study is that it will inform an RCT that doesn’t have these shortcomings.

Following the 2011 study the device was superseded by the AiperMotion 500, which was smaller and lighter, had a longer battery life and a colour screen, provided anthropomorphic-visual motivational feedback and allowed the user to make retrospective ‘corrections’ to calorie and physical activity data. In accordance with the Medical Research Council’s (MRC) Complex Intervention guidelines [16] we planned a feasibility study to determine the acceptability of the AiperMotion 500 and the feasibility of conducting an RCT of the intervention in the UK, as a first step to more robust evaluation of the effectiveness of the intervention. The manufacturers were aware of the project but were not involved.

## Methods

### Research aims and objectives

The primary aim of the study was to investigate the feasibility of conducting a future mixed-methods RCT of an mHealth supported weight loss intervention (AiperMotion 500) for overweight/obese individuals with T2DM. The primary objectives were to include measures to address the above noted shortcomings of other studies, review operation of the study (especially including recruitment, randomisation procedures, and attrition) and explore participants’:

- Views of the education on diet, exercise and use of the technology;
- Views on acceptability of the technology;
- Adherence to using the technology;
- Perception of how motivational the technology was;
- Experience of taking part in the study.

The measurement of weight loss and change in HbA1c and inflating participant numbers allowed a secondary aim of obtaining an initial indication of any weight loss and HbA1c trends. Thus the secondary objective was to:

- Compare mean weight loss and HbA1c change across the study arms using descriptive statistics.

### Design

The mixed methods feasibility study occurred during April–December 2013 (the maximum time available from funding), 5 weeks setting up the study ending in launching public awareness of the study, 5 weeks for recruitment (incorporating the training sessions), 20 weeks for participation and, 9 weeks for analyses and write up. For the feasibility study each participant was asked to take part for 12 weeks aiming at weight loss and blood sugar reduction followed by 8 weeks of maintenance of their achievement (reflecting the goal of achieving and observing a longer term change in behaviour and maintained weight loss/blood sugar reduction [17]). These durations were judged sufficient for the primary objectives and minimally sufficient for the secondary objectives. Longer durations for both goals of reduction and maintenance in any future trial were anticipated but were beyond resource and duration limits of the feasibility study.

Participants were randomly allocated to one of three groups:

- Group One-advice on diet and exercise;
- Group Two-as Group One plus use of AiperMotion 500;
- Group Three-as Group Two plus email motivational support (to echo other AiperMotion studies [15]).

For the purposes of the primary study objectives methodologically recruiting around ten participants in total could be seen as sufficient, but for the secondary aim and objective more would be needed. About ten participants in each arm was judged as minimally sufficient, but to account for high published attrition rates (47–60 %) [18, 19] twenty in each arm was selected as a suitable ambition.

### Intervention

**Fig. 1:**
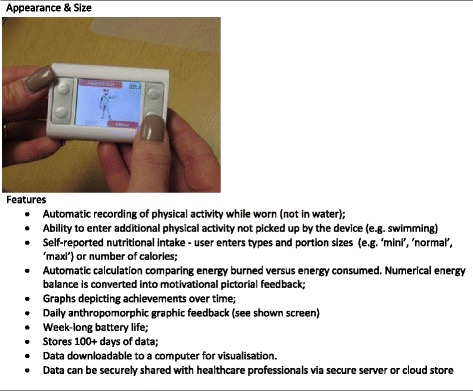
Features of AiperMotion 500

The AiperMotion 500 is a wearable device, preferably worn at the hip (with less accuracy it can be carried in a pocket or bag). Added motivation to lose weight occurs through user awareness of the device’s automatic recording of physical activity, the act of recording nutritional intake for eating healthily, and receiving graphical and pictorial feedback on these measures (both immediately and over time). The features of the device are outlined in Fig. 1.

For Group 3 further motivational impact may have occured through emailed sending of weekly event diaries and receiving motivational feedback in return.

### Procedures

At the beginning of the 12 week intervention all participants received 90-min group training, delivered by the research team, around appropriate behaviours to lose weight and control their HbA1c, specifically:

- Eating a low Glycaemic Index (GI) diet (this included the first hand experiences, tips on cheap information sources and ingredients, and tasting of example baked foods with low GI);
- Maximising physical activity;
- Neuro-Linguistic Programming (NLP) was also employed as the primary motivational component of the training to enable participants to visualise themselves at their ideal weight and then set goals to reach it. NLP relies on making an individual aware of their subconscious sensations, movements, language and perceptions (as well as their conscious ones), and using this to explore alternative ways of thinking about their hopes, behaviours and experiences [20].

All participants were given a booklet summarising the training. While participants were encouraged to try the low-GI diet they were told they could choose to follow their preferred diet if it had been previously successful. The recommendation regardless of the selected diet was to combine this with increased physical activity. Participants were also told they could individually choose their own weight-loss goal to increase motivation.

Randomly arranged pre-sealed envelopes were used to randomly allocate individual participants to study groups; the envelopes were held by a responsible person who was physically remote from the training and independent from the study. At the end of training one of the researchers contacted the responsible person, who opened the next envelope and over the telephone revealed the next participant’s allocation. Allocation was concealed from participants and researchers until training was completed.

Groups Two and Three then received a further 60 min training in use of the AiperMotion 500 during which their individual characteristics (weight, height, gender and age) were entered into their devices. These participants were asked to wear the device during waking hours and to report the amount of time that they engaged in physical activity where the device was not worn (e.g. swimming). They were also asked to enter dietary information into the device whenever they consumed food or drink (apart from water). They could choose to enter exact calorific values or category of meal and portion size. The motivational feedback was also highlighted to them. Participants in Group Three were asked to send weekly emails to the research team describing any positive or negative events which had impacted their conformance with the study or motivation to lose weight, and any behaviour changes, during the previous week (‘Event Diaries’). Emailed replies delivered tailored positive encouragement—thus potentially adding further positive motivation. If an Event Diary was not received then a reminder was sent to the participant.

Table 1 provides an overview across the three arms and individual participant’s entire timelines of their exposure to training, study measures and data collection. Each participant’s five visits should accumulate to about 280 min. As can be seen this involved a combination of quantitative and qualitative measures to address the research aims including those of the anticipated future trial.

**Table 1:** Participant activities at research venue visits

In week	Actions/Events	Group 1	Group 2	Group 3
1	Answer baseline questionnaire	✓	✓	✓
	Collection of blood samples for HbA1c	✓	✓	✓
	Measure height and weight	✓	✓	✓
	Training on diet and weight loss	✓	✓	✓
	Randomisation	✓	✓	✓
	Training on use of the device	–	✓	✓
	Training on email support	–	–	✓
6	Measure weight	✓	✓	✓
	Collection of blood samples for HbA1c	✓	✓	✓
	Download data from device	–	✓	✓
12	Measure weight	✓	✓	✓
	Collection of blood samples for HbA1c	✓	✓	✓
	Answer repeat questionnaire	✓	✓	✓
	Download device data	–	✓	✓
	Interviews/focus groups	–	✓	✓
16	Measure weight	✓	✓	✓
	Collection of blood samples for HbA1c	✓	✓	✓
	Answer repeat questionnaire	✓	✓	✓
	Download device data	–	✓	✓
	Interviews/focus groups	–	✓	✓

As can be seen in Table 2 the 12 week intervention was followed by a weight maintenance period. Originally intended to be 8 weeks in length, due to slower and lower recruitment than aimed for, the investigators reduced this to just 4 weeks (so that 4 further weeks could be spent recruiting more participants to address the secondary objective within the available duration). Group 3 Event Diaries and associated motivational emails were stopped during the maintenance period; but all participants were asked to continue using their device. Table 2 summarises the participants’ intended activities over the 16 weeks.

**Table 2:** Participant aims and planned use of interventions during their participation

Timeframe	Participant aims and interventions	Group 1	Group 2	Group 3
Weeks 1–12	Aim to reduce HbA1c and weight	✓	✓	✓
	Follow their choice of diet	✓	✓	✓
	Follow their choice of exercise	✓	✓	✓
	Measure their weight regularly	✓	✓	✓
	Adhere to use of device if available	–	✓	✓
	Send & receive emails weekly	–	–	✓
Week 13–16	Aim to maintain HbA1c and weight	✓	✓	✓
	Adapt their diet as necessary	✓	✓	✓
	Adapt their exercise as necessary	✓	✓	✓
	Measure their weight regularly	–	✓	✓
	Adhere to use of device if available	–	✓	✓
	Send & receive emails weekly	–	–	–

### Use of the device

It is useful to also describe the expected use of the device the participants would make during the study. The device starts a new record for each calendar day at midnight. Participants would start by putting the device onto their clothing (ideally at the hip, but otherwise elsewhere, but if really necessary putting it into a bag they carry). The device immediately starts to record their physical activity automatically. If they wish they can alter their physical activity goals for that day. Similarly the goal daily calorific reduction can be set, or change in weight recorded. All of these values would be anticipated to be changed only occasionally—e.g. when the user wants to reset their goals.

After each occasion they have eaten or drunk something they make a calorific entry into the device—choosing whether they will use the quick estimated choices of type and size of meal, or to calculate the exact calorific value and enter that. The former choices are calibrated to the user’s BMI, so nominally entering an appropriate number of ‘normal’ sized selections would lead to no weight change, ‘small’ and ‘large’ leading to weight loss and gain respectively.

If they need to remove the device from their clothing at any time then they can add a calorific value to the exercise record. The device allows them to select a type of physical activity and its duration—making an estimate of the energy burnt based on their BMI.

At any time they can obtain motivational feedback that is based on food intake, energy burnt and their BMI. On the screen they can see an anthropomorphic figure and get a sense of how they are doing that day, or they can choose to view the results and examine the calorific values of exercise and food and the energy balance, or look at how much of the required time for their exercise goals they have achieved, for that day or for up to the preceeding 7 days. More graphical visualisation of the device-collected data can be viewed by them downloading the data to software on their computers where longer periods of time can be reviewed.

### Outcome measures

#### Quantitative

Participants completed a questionnaire at baseline, at the end of the intervention, and at the end of maintenance. The questionnaire [see Additional file 1] recorded:

- Demographic information including : at baseline age, gender, height, education, employment status, use of ICT; on all occasions living circumstances, medical history, medications, willingness to change, notification of any change in circumstance;
- Health behaviours, on all occasions, such as dietary habits, physical activity, smoking behaviour, and alcohol intake.

Participants visited the NIHR Clinical Research Facility (CRF) at the Royal Hallamshire Hospital in Sheffield at weeks 0, 6, 12 and 16. During these visits weight (kg) was measured using a SECA medical class III body weight scale, and height was checked at baseline using a wall-mounted stadiometer. A blood sample was taken at each visit to assess HbA1c. Device data on dietary intake, physical activity and data entry time logs were downloaded at week 6, 12 and 16. The research team maintained trial management records in which levels of participation and attrition (including any volunteered reasons for withdrawal) were logged.

#### Qualitative

Qualitative focus groups and (where participants preferred it) individual interviews were conducted with a convenience sample of participants from Group Two and Three to explore perceptions of the technology’s acceptability and motivational value, and experiences of being involved in the study. The Technology Acceptance Model (TAM) for Mobile Services [21] informed development of the interview guide on issues relating to the device; inspiring questions/topics for discussion based around the: value to users, ease of use, trust (including perceived reliability), and ease of adoption. To add to this participants were asked for their views on the education/training session at the start of the study and were invited to share comments/views on any other relevant topic. Interviews and focus groups were audio-recorded and transcribed verbatim.

### Participants

Overweight and obese members of the public in South Yorkshire with T2DM were recruited through advertising via the volunteer database for The University of Sheffield, Facebook, posters in public places, local newspapers and third sector organisations. Interest was indicated by telephone or email after viewing the study’s website (which included the participant information sheet). They were then invited to attend a one on one introductory session at which written informed consent was collected and a training date booked. Participants were offered £10 cash per visit as nominal reimbursement (which was not declared in the advertisements).

The eligibility criteria for the study were as follows:

- Inclusion criteria:
  - Diagnosed with T2DM;
  - Aged 30–60 years;
  - Body Mass Index (BMI) between 25 and 40;
  - Aiming to lose 5 % or more of body weight through managing diet and exercise;
  - Willing to use Information Technology (IT);
  - Able to communicate and read in English (limitation of the technology).
- Exclusion criteria:
  - Pregnant, planning to become pregnant, or had been pregnant within 6 months of the study starting;
  - Taking medication which affects weight, e.g. antidepressants;
  - Diagnosed with a chronic condition other than T2DM, including mental health conditions;
  - Used computer software, a website or mobile app within the last 6 months to reduce weight or blood sugar;
  - Unwilling to use IT;
  - Cognitive or other significant impairments which impede ability to participate or give informed consent.

The inclusion and exclusion criteria were similar to those used in Luley’s RCT with AiperMotion 440 [15], with the exception that pregnancy was a reason for exclusion but vegetarianism was not, and although T2DM diagnosis was a requirement, heightened HbA1c or regular use of antidiabetic medication was not. Furthermore, participants in the current study were permitted to use any weight loss approach provided they were not delivered using mHealth or other remote communication technology, echoing the RCT from Haapala [22]. Clinical experience and evidence [23] suggested the 30–60 age range as potentially more receptive than older ages to the use of digital technology to influence health. The goal was 60 recruits (20 per arm) which exceeds that usually employed to observe any major issues regarding the feasibility of a methodology and/or an intervention, but offers the potential for initial insight of the clinical changes due to the intervention and pre-empts possible high attrition rates seen in previous studies on obesity (47–60 %) [18, 19].

### Data analysis and statistics

Frequency of wearing the device (i.e. to monitor physical activity) and frequency of entering dietary information were used to provide an indication of participants’ adherence to use of the device. Qualitative data were categorised into a priori and emerging themes in accordance with Framework Analysis [24]. Descriptive statistics were used to summarise weight and HbA1c changes across the groups and timeframes.

## Results

Results are presented following the order of the Primary and Secondary research objectives.

### Primary research objectives

#### Recruitment to the study

As there was a requirement for participants to have IT literacy, visits to the study website provide an indication of public awareness and interest in the study. The website in a 2 month period (when visit data were available) had 210 unique visits and 524 page views. 38 % of visitors viewed pages beyond the home page. Table 3 summarises the visits to individual pages, omitting the home and index pages and any not visited at all.

**Table 3:** Study website statistics over 2 months

Web page	No. of visits
Eligibility	47
The Study	46
Volunteering	45
Study Details	35
Volunteering Details	33
Detailed Eligibility	33
Contact	22

While researchers reported that at least 85 people enquired about the study, the logs of telephone enquiries indicate 65 attempts to be recruited. Table 4 summarises the recorded reasons that were identified to exclude participation.

**Table 4:** Reasons for exclusion of the 38 enquirers not recruited to the study

Reason for exclusion	No. of enquirers
Not diabetic	6
Too old or too young (respectively)	6 + 1
BMI too high or too small (respectively)	7 + 1
Unwilling or unable to use IT	2
Taking weight—affecting medication	2
Other serious medical condition	2
Cannot attend the required visits to research facility	4
No reasons recorded/failed to establish dialogue	14

The recruitment rate was slower than anticipated. Recruitment was extended by 4 weeks (and correspondingly the maintenance period was shortened to 4 from 8 weeks). The study was advertised in May, June and July while recruitment occurred during June and July. To increase recruitment, inclusion criteria were also relaxed to include Type 1 Diabetes and ages up to 65, resulting in seven extra recruits.

Twenty-seven participants were recruited to the study (nine to each Group). These participants became aware of the study as follows: fifteen via emailed invitations to consider participation; three from third party online promotion of the study; two via newspaper adverts; two from posters displayed in public venues; and five via word of mouth.

A summary of participants’ demographic characteristics at Baseline is shown in Table 5. Although participants diagnosed with depression or receiving prescribed anti-depressant medication were excluded from the study, two made it into the study as they had not revealed this information when recruited.

**Table 5:** Demographics of Study Participants at Baseline^a^

Characteristic	Group 1 (*n* = 9)	Group 2 (*n* = 9)	Group 3 (*n* = 9)	Total (*N* = 27)
Age in years, Mean (SD)	55.7(9.9)	52.1(7.8)	50.8(8.1)	52.9(8.6)
Male	0(0)	5(55.6)	7(77.8)	12(44.4)
Female	9(100)	4(44.4)	2(22.2)	15(55.6)
Ethnicity				
British	7(77.8)	8(88.9)	8(88.9)	23(85.2)
Irish	1(11.1)	0(0)	0(0)	1(3.7)
Asian	0(0)	1(11.1)	0(0)	1(3.7)
African	1(11.1)	0(0)	0(0)	1(3.7)
Caribbean	0(0)	0(0)	1(11.1)	1(3.7)
Living Circumstances				
Living with others	7(77.8)	8(88.9)	6(66.7)	21(77.8)
Living alone	2(22.2)	1(11.1)	3(33.3)	6(22.2)
Socio-Economic Status				
Professional employment^b^	3(33.3)	2(22.2)	7(77.8)	11(40.7)
Home owned outright	4(44.4)	2(22.2)	3(33.3)	8(29.6)
Mortgage	1(11.1)	5(55.6)	5(55.6)	12(44.4)
Rented	4(44.4)	1(11.1)	1(11.1)	6(22.2)
Physical Activity & Diet History				
Physical Activity: Never/Rarely	4(44.4)	4(44.4)	3(33.3)	11(40.7)
: Sometimes	3(33.3)	4(44.4)	5(55.6)	12(44.4)
: Often	2(22.2)	1(11.1)	1(11.1)	4(14.8)
Tried dieting in the past	9(100)	5(55.6)	7(77.8)	21(77.8)
Weight loss success	8(88.9)	6(66.7)	7(77.8)	21(77.8)

^a^Data are represented as the number (percentage) of study participants unless stated otherwise

^b^Implying level of education and current and/or past as in socio-economic profiling

#### Randomisation

Randomisation took place in the group setting at the end of training. One participant became distressed upon finding out that they had been allocated to the control group, exacerbated by being told within the training group setting, which in turn also hampered the research team’s ability to support the participant. This issue was subsequently managed by segregating participants into study arm groups before they were told their allocation.

Although the allocated Groups were comparable on most demographics, there were significant differences across gender and socio-economic status indicators. For example, there were no males and more participants renting accommodation in the control group, and a greater proportion of professionally employed participants in Group 3. However these baseline demographic differences between groups can be attributed to the small sample size.

No other problems were encountered with randomisation.

#### Attrition and loss to follow up

During the 16 week study out of the 108 (i.e. 27 × 4) total planned participant visits to the research venue 94 were achieved. By the end of the study five participants were lost due to attrition, two more were lost to follow up, together this amounts to 26 % of the sample. Of these, five withdrew prior to the end of the 12 week intervention period (Group 1: 2; Group 2: 1; Group 3: 2), and a further 2 participants were lost to follow up from Group 3 in the weeks 12 and 16 period. The exit questionnaire [see Additional file 2] was sent to those withdrawing, and ill health or moving out of the area were volunteered as reasons for withdrawal. However one also mentioned too frequent visits and parking being a problem at the research venue. No one mentioned problems or issues with use of the Aipermon 500 device or with the training.

#### Qualitative analysis results

Group Two and Three participants took part (6 males, 3 females) in qualitative interviews and focus groups. Qualitative data were coded into the following naturally emergent themes (unsurprisingly some of which correlated with those from the TAM for Mobile Services):

- Diet, exercise and device training (cf education)
- Ease of adoption (cf acceptability)
- Trustworthiness (cf acceptability)
- Ease of use (cf adherence)
- Value (cf perception of motivation)

The above list highlights the match to the primary research objectives shown in brackets. The following results sub-sections report illustrative and specific results relating to training, acceptability, adherence, and perception of motivational value (as shown above). Any significant additional information available from the execution of the study is also reported. The final primary research objective of experience of taking part in the study is reported at the end.

#### Diet, exercise and device training

The education/training sessions around diet and exercise, and use of the device, generated mainly positive comments but with a few negatives, e.g. :

*“I found it very good ….. I was left in no doubt as to the importance of the diet …. and the types of food you should be avoiding”. P1/018*

*“I was confident yes (to use the device after training) … I went in (the device) and went home and put all the data in and then put it on the following day”. P1/023*

*“Confident to use the device after training?, No”. P1/020*

The exercise training, while mentioned in the introduction in the focus groups, was not a specific sub-topic—although there was an open opportunity to comment. There were no comments specifically about the exercise training, positive or negative, but there were comments indicating doing more exercise (most often with reference to motivation attributed to the device):

*“I constantly use the stairs so yes it has made it different. I think about walking instead of riding”. P1/023*

*‘I’m just far more active’. P1/009*

Receiving a booklet with the training information in it, the signposting to low-GI diet guides, and practical knowledge of one of the trainers in making low-cost low-GI selections and baking, were appreciated. Two participants had minor problems with device training and required extra practice during the training session.

A few individuals disliked the NLP element of the training. It asked participants to visualise themselves at their ideal weight, a task which some participants found upsetting as they felt the exercise and its implied weight loss were unrealistic.

#### Acceptability

Three participants did seek (and were given) help in instances where they wanted to correct for errors they had made when entering exact calorific values. The focus groups mainly suggested that the device was easy to incorporate into their everyday lives, although problems were reported:

*“Certain clothes that I wore it was difficult to keep on…a skirt or more dressy trousers it would dig in…that’s nothing compared to the benefits”. P1/009*

In another case a participant adapted how they carried the device even though it was not in the preferred location for accuracy:

*“I usually keep it in my bag…I don’t think anybody thinks (anything) about it you know, it is just there”. P1/005*

Opinions were mixed, too, on how trustworthy the data in the device were:

*“Whatever you put into it is accurate…but it’s whether your (calorie) counting is accurate”. P1/012*

*“What it said on the machines (at the gym) was not necessarily what was registering (on the device)”. P1/012*

There were 3 instances of data being lost due to faulty or lost devices—creating some gaps in the data collected.

#### Adherence (to using the technology)

The focus group participants reported the device was easy to use, in some cases linking it directly to adhering to increased exercise or dieting :

*“It is quite a simple straightforward device and the size is not that intrusive… I found it extremely easy (to enter information)… I found it fine, no problem at all”. P1/008*

*“I’ve used it so to me that shows my interest is high, otherwise it would be sitting on the side and it would be unused”. P1/009*

*“I think it’s made me really positive, I really do watch what I’m doing and I do get cross if I put it on and I’ve not done loads of steps (laughs)”. P1/023*

There were also some comments implying summer holidays may have interfered with adhering to use of the device and in following their chosen diet and physical exercise programme.

An objective and quantitative perspective on adherence is added through considering the days the device was worn and days on which diet was entered. Adherence has been simply calculated as: the number of days the device had been worn divided by the total number of days participants had the opportunity to wear the device; and, the number of days on which at least 950 cal of nutritional information were inputted divided by the total number of days participants had the opportunity to input nutritional information. The threshold of 950 cal was adopted as representing a minimal level of nutritional intake on a given day.

Table 6 shows the levels of adherence to the use of the device in this study. These adherence averages are calculated including ‘missing data’ when participants were unable to provide the daily monitoring data (due to their devices being lost or broken/faulty). Across the 16 weeks four participants in Group 2 and another four in Group 3 wore their devices for in excess of 90 % of available days; the days recording their diet for the participants in these sub-groups averaged to 87 and 81 % respectively. Based on the above definition there was generally greater adherence to wearing the device than to inputting nutritional information.

**Table 6:** Adherence to using the Device by Participants with collected Weight measurements

Weeks	Group 2			Group 3		
	*n*	% worn^a^	% diet entries^b^	*n*	% worn^a^	% diet entries^b^
1–6	9	62	62	8	61	58
7–12	8	65	59	7	69	70
13–16	8	75	49	5	94	70

^a^Percentage of days worn from total days of access to the device

^b^Percentage of days of nutritional data of at least 950 cal was entered from total days access to the device

Group 3 participants were also asked to submit weekly emails from which they would receive motivational feedback. Adherence to this over the 12 weeks it operated was low at an average of 31 % (3 participants supplied 7–9 emails, 2 supplied 3 or 4, and the remaining 4 supplied 0 or 1). Those who supplied 0 or 1 emails correlated exactly with those who withdrew or were lost to follow up in Group 3.

#### Perception of motivational value

In addition to the quotations from P1/009 to P1/023 in the preceding Adherence section there were further indications of positive motivational value of the device:

*“Getting the cup, the motivation it gives you that’s the biggest plus of it all, it motivates you in a way that nothing else does because it’s constantly measuring what you’re doing”. P1/009*

*“Having the device does make you think and do things differently…my sugars have been the best they’ve ever been”. P1/026*

But some reported disadvantages or negative experiences (e.g. in comparison with a face-to-face intervention):

*“It has not really (changed my behaviour), I’ve been a bit stressed and moody…it is to do with other things”. P1/005*

*“I might have been better with a trainer or a coach”. P1/026*

There were also mixed comments about the weekly email dialogues :

*“keeps you going”. P1/001*

*“I found it amusing to get these support emails ….. if people take an interest in you and your results then that can modify one’s behaviour” P1/020*

*“I haven’t religiously sent the emails …. If I didn’t have anything positive to say I didn’t really want to send the email (laughs)”. P1/018*

#### Experiences of involvement in the study

Participants reported that they felt that the study staff were always on hand and very helpful. Attendance at the research facility at agreed dates and times was not perfect. There were several occasions where agreed visits had to be re-arranged—usually because of a failure to arrive rather than a planned re-arrangement. Participants in most cases of both planned and unplanned re-arrangements gave reasons based on being too busy or another commitment.

With the range of BMI of recruited participants there were a couple of those with higher BMI who felt uncomfortable with the presence of people at the lower end of the study BMI range. The latter were mainly people with Type 1 diabetes.

As the study was not funded or approved to take advantage of the technically possible uploading of data to a third party secure online server a more manual process had to be adopted. Participants had to hand over their devices during research facility visits. The data would then be downloaded by the researchers, which proved cumbersome. However, some participants had a worse experience because they had completely downloaded the data from their devices to their computers at home. This made the process much more difficult for the affected participants as they had to be given instructions for copying the required folders and files to a memory stick to bring to the visits to the research facility. With occasional delays requiring extra support this was still completed successfully with the exception of those participants lost to follow up.

Focus group participants were generally satisfied with their experience of being involved in the study, and many stating that they enjoyed using the intervention and were satisfied with the technology. A few expressed difficulties with keeping to their chosen diet or dissatisfaction with having to calculate calories to then enter into the device. Some of these reported converting to using the device preset meal types and sizes instead and ended up being satisfied like those who had chosen to use this method early on—all participants using this method found it easy and acceptable. A majority expressed a willingness to take part in future studies if possible.

### Secondary research objectives

#### Weight and HbA1c

Tables 7 and 8 shows that participants in Groups 2 and 3 (with the device) had greater reductions in weight and HbA1c compared to Group 1 (without the device). Inferential analyses were not conducted as the sample sizes were small, thus this trend should be interpreted with caution.

**Table 7:** Summary of Means, Ranges, and Changes from Baseline in Weight

	Group 1				Group 2				Group 3			
	Baseline	Wk 6	Wk 12	Wk 16	Baseline	Wk 6	Wk 12	Wk 16	Baseline	Wk 6	Wk 12	Wk 16
	(*n* = 9)	(*n* = 8)	(*n* = 7)	(*n* = 7)	(*n* = 9)	(*n* = 9)	(*n* = 8)	(*n* = 8)	(*n* = 9)	(*n* = 8)	(*n* = 7)	(*n* = 5)
Weight (kg)												
Mean	98.6	100.0	101.2	101.1	95.8	93.1	89.5	89.1	102.0	97.8	99.1	95.1
Range	83.0–110.8	83.0–111.4	83.4–111.8	84.0–110.4	74.2–123.0	73.0–121.0	73.5–108.2	73.6–108.6	91.2–113.4	87.2–108.2	85.2–110.4	84.2–103.6
Mean change from Baseline	–	+0.1	+0.8	+0.7	–	−2.7	−2.9	−3.3	–	−3.0	−3.2	−3.0
Range change from Baseline	–	−1.0 to +1.6	−0.2 to + 2.1	−0.6 to +2.0	–	−5.0 to–1.2	−7.4 to–0.7	−8.8 to–0.6	–	−8.6 to +0.4	−12.0 to +2.0	−13.0 to +3.0

**Table 8:** Summary of Means, Ranges, and Changes from Baseline in HbA1c

	Baseline	Wk 6	Wk 12	Wk 16	Baseline	Wk 6	Wk 12	Wk 16	Baseline	Wk 6	Wk 12	Wk 16
	(*n* = 9)	(*n* = 7)	(*n* = 6^a^)	(*n* = 7)	(*n* = 9)	(*n* = 8)	(*n* = 8)	(*n* = 6^a^)	(*n* = 9)	(*n* = 8)	(*n* = 6^a^)	(*n* = 3^a^)
HbA1c (mmol/mol)												
Mean	60.4	60.9	64.3	63.6	57.6	54.0	50.6	49.2	65.8	58.6	57.7	46.3
Range	44.0–106.0	44.0–106.0	40.0–107.0	43.0–101.0	44.0–78.0	42.0–81.0	36.0–66.0	38.0–63.0	48.0–115.0	45.0–115.0	41.0–79.0	43.0–53.0
Mean change from Baseline	–	−0.9	0.0	+0.9	–	−4.8	−8.1	−10.7	–	−2.9	−14.8	−5.0
Range change from Baseline	–	–9.0 to +5.0	–4.0 to +3.0	–5.0 to +9.0	–	–12 to +3.0	–13.0 to 0.0	–26.0 to 0.0	–	–10.0 to 0.0	–39.0 to–2.0	–12.0 to +2.0

^a^Either one or two participants’ results being missing due to sample handling errors (not withdrawal)

## Discussion

This study was the first attempt to independently evaluate the acceptability and potential effectiveness of the AiperMotion 500 in a UK setting. Overall the device was well-liked and perceived to be acceptable, motivational and easy to use by most participants. Good average levels of adherence with wearing the device and inputting nutritional data were observed (NB faulty or lost devices created gaps in the device data which in turn reduced the averages reported in Table 6), supporting previous studies which show that providing ‘real-time’ feedback in self-monitoring is valued [25]. However, a certain amount of polarity in adherence of individuals to using the device was evident. With more detailed observation any polarity in use of mHealth to monitor physical and dietary activity by individual participants might be investigated in future pre-trial studies. Although caution should be employed in interpreting the quantitative results of the study, the mHealth intervention does show promise in helping people with T2DM to reduce their weight and HbA1c levels. The email motivational feedback, while possibly causing higher adherence to use of the device, did not appear to make a difference to the amount of weight loss and HbA1c reduction observed, although again caution should be employed.

Increased motivation attributable to the use of the device is of course an important factor in considering the results presented here. In reality the added motivation occurs within a context—including many influences e.g. the participants’ routine environment, life events, and experiences from other elements of the study. There was no intent to separate or directly observe the motivation from the device. Beyond participants’ comments the study evidence can only give inferences about motivational impact indirectly from the results on adherence, acceptability, attrition, and from the weight and HbA1c changes. These results also broadly imply a useful and positive motivational impact.

Recent systematic reviews demonstrate the potential of portable technology for weight reduction in adults [26–28]. However there is an acknowledged lack of rigorous RCT evidence in this area [26, 27], as well as uncertainty around the effectiveness of mHealth compared to other weight loss interventions [26], whether any benefits are sustained long-term [27, 28], which components of mHealth are most effective [29], which populations should be targeted [26], and whether they would be technically and financially viable for use on a larger scale [27]. The current feasibility study is an important first step towards answering some of these questions for weight loss in the T2DM population, and will be used to inform the design of a future RCT.

Problems were discovered with some of the practicalities of conducting the study, including recruitment and randomisation, inclusion/exclusion criteria, length of maintenance period and nature of the education session. An attrition rate and loss to follow up of 26 % is still a concern and rather than one significant cause there seems to have been a number of contributory factors for the participants in this study, including traumatic events/illness in the family, busy lives, frequency/density of visits to the research facility, and difficulties with parking at the research facility. Due to time and budgetary constraints, only 27 of the target 60 participants were recruited; even after attrition and loss to follow up those remaining were still sufficient to inform the feasibility of operating a future RCT.

The results demonstrated that it is feasible to conduct an RCT investigating the effectiveness of an mHealth device in aiding weight and HbA1c reduction in T2DM (with a view to conducting a large multi-site RCT), assuming that modifications to the methodology are made:

1. The inclusion and exclusion criteria should be less prescriptive so that people over the age of 60, people with co-morbid depression, and people with other co-morbid long term conditions, are included. Some older T2DM patients will be interested in using technology to manage their condition [30] and open to use of digital technology [23]. There is a high rate of co-morbid depression [31] and other co-morbid long term conditions [32] among people with T2DM. It might be argued, despite some individuals on anti-depressant medications experiencing weight gain [33], that they could still benefit. Including such participants may require special allowances in the protocol.
2. The study duration should be substantially increased to include longer periods of weight/HbA1c reduction and maintenance, in order to determine the long term effects of using this intervention. Twelve weeks was short for the ultimate weight loss goals of many of the participants. Although not explored at the time of the study, such a device as the Aipermon 500 (i.e. the intervention used here) would be well suited for a user to aim for an energy balance of zero during weight maintenance.
3. The randomisation process should take place in private rather than in a group setting. Support should be in place if participants become distressed by their allocation, and ideally all participants should be offered the opportunity to use the device after the study has completed (although this would increase the cost of an RCT).
4. Any additional motivational techniques to encourage adherence should be of known value in weight loss and/or diabetes; where any uncertainty exists (perhaps as with NLP) they should be evaluated separately.
5. Participants should be recruited through diabetes clinics and other healthcare sources, rather than through public advertisements alone, in order to access the T2DM population pool more directly. It may be useful to note that after the study an NHS formed public involvement group reviewed the recruitment materials (to inform what should be used for the future RCT). Their view was that the information on the study website pages was entirely sufficient and that the ethically required detailed Participant information sheets were unnecessary, if not off putting.
6. In light of the slow recruitment and failure to reach the target sample size the previous modifications on their own may not be enough. Thus some further measures to improve recruitment should be considered, e.g. a longer period to recruit. Financial incentive is another. Simply advertising and paying participants to take part, even if linked to just making sure they adhere to the administrative basics of the study, may increase recruitment without undue impact on outcomes. Financial motivation tied to individual participant success during a study has been shown to be effective as an intervention in its own right; however in the longer term follow up of the benefit is also reported to be eroded [34].
7. Careful consideration should be given to the location of any visits participants make for the study. For instance, use of a health service facility as a location to collect blood samples seems appropriate but if at all possible there should be easy parking at and travel to the location. Use of local clinics, GP surgeries or other places where the participant’s journey is shortened and made easy may help to reduce attrition.
8. People are not homogeneous in their confidence or capability to use ICT. Despite having reported ease of use, having on request responsive individualised support to using any technology during the early weeks of the study (i.e. period of adoption of the device) may help both with adherence and reduce attrition. As a minimum offering video instructions for participants to access at home (rather than just printed material) should be considered.
9. Including explicit opportunities for participants to report faulty or lost devices may not only improve data collection but, similarly to the previous modification, also improve adherence and reduce attrition.
10. More blinding measures should be included, for instance so that any staff taking physiological readings are not aware of a participant’s allocation [12].
11. Approval should be sought to allow the automatic transfer of data from the device to a secure server which can be seen and downloaded by the research team and the healthcare professional(s) responsible for the individual’s care. This would mean fewer visits to the CRF, resulting in less chance of attrition, loss to follow-up and loss of data, and greater convenience for participants.
12. Adherence in this study was calculated based on overall percentages for wearing the device and inputting of nutritional values. Use of simple convenient estimates of adherence is not untypical for similar published studies. However this neither considers the voracity of the collected data nor the true complexity of adherence to behaviour change interventions [35]. In a future RCT it may be more appropriate to consider adherence as a ‘specific’ measure to individual patients [35] before considering ways to judge adherence across a trial.

A pilot RCT, followed by a full scale RCT, with suitably amended protocols are required to evaluate the promising trends identified in the feasibility study and to provide a robust indication of effectiveness. Unfortunately, since the feasibility study’s completion, the AiperMotion 500 has been withdrawn as its manufacturer stated it wished to invest its resources in other health product lines. Uncertainty around willingness of individuals or health services to purchase the technology, especially with burgeoning competition from smart phone fitness apps, may have contributed to this decision. At the time of the study the AiperMotion 500 was unique from many other mHealth interventions due to the combination of its characteristics: CE marking as a medical product, anthropomorphic pictorial feedback; flexibility to allow the user to enter either estimated or specific calorific values for food consumed; allowing input of additional exercise when the device was not worn; and in allowing secure cloud or server-based sharing of individual patient data with healthcare professionals. Features of the AiperMotion 500 were highly valued and therefore ideally an app or device incorporating similar features needs to be found or developed in order to continue evaluation of this intervention. However it is noted that a meta-analysis of digital interventions for T2DM suggests feature variation between interventions has only minor effects on blood glucose results [36], so there is room for some variation in features in a future version of the intervention.

## Conclusions

The feasibility study has been a valuable first step towards informing evaluation of the effectiveness of an mHealth-based complex intervention for weight loss/HbA1c reduction in T2DM, in accordance with the MRC framework [16]. Methodological lessons will be carried forward into an RCT of a similar intervention but may also more generally be considered in similar feasibility studies of other mHealth technologies for obesity and/or diabetes. An intervention based on technology with similar features to the AiperMotion 500 is a potentially acceptable and promising aid for individuals with T2DM to control their HbA1c and weight.

## Abbreviations

BMI, body mass index; CE, European Conformity; CLAHRC SY, Collaboration for Leadership in Applied Health Research and Care for South Yorkshire; CRF, Clinical Research Facility; GI, glycaemic index; HbA1c, (make sure this is defined in the text as well); IT, Information Technology; mHealth, mobile health; MRC, Medical Research Council; NLP, Neuro-Linguistic Programming; NHS, National Health Service; NIHR, National Institute for Health Research; RCT, Randomised Controlled Trial; T2DM, Type 2 Diabetes Mellitus; TAM, Technology Acceptance Model; UK, United Kingdom

## Additional files

Additional file 1: In the file called ‘Additional File 1’ can be found the study’s “A feasibility study of weight loss interventions in people with diabetes Baseline Questionnaire”. It contains the questionnaire that was used to provide participant descriptive data at the baseline but also indicates the data that was collected in repeat questionnaires at the end of both weight/HbA1c reduction and maintenance periods. (PDF 324 kb) Additional file 2: In the file called ‘Additional File 2’ can be found the study’s “Weight loss amongst patients with Type 2 diabetes—Exit Questionnaire” it was an optional questionnaire sent to participants who elected to withdraw from the study. Used to identify reasons for withdrawing. (PDF 238 kb)
