# Supplementary material for: Feasibility study of portable technology for weight loss and HbA1c control in type 2 diabetes
Source: BMC Med Inform Decis Mak. 2016 Jul 15;16:92. doi: 10.1186/s12911-016-0331-2 (PMC4946211; doi:10.1186/s12911-016-0331-2)
Supplement: Additional file 1: — In the file called ‘Additional File 1’ can be found the study’s “A feasibility study of weight loss interventions in people with diabetes Baseline Questionnaire”. It contains the questionnaire that was used to provide participant descriptive data at the baseline but also indicates the data that was collected in repeat questionnaires at the end of both weight/HbA1c reduction and maintenance periods. (PDF 324 kb) [file 12911_2016_331_MOESM1_ESM.pdf]

ID number ☐☐/☐☐☐

A feasibility study of weight loss interventions in people with diabetes

Baseline Questionnaire

Remark : Questions repeated in visits after baseline are shown in red

Date of completion ☐☐ ☐☐ ☐☐☐☐

Socio-demographics

1. Gender ☐ Male ☐ Female

2. Age ☐ years

3. Live with others ☐ Yes ☐ No

Live with…… ☐ Spouse/ partner ☐ Child/ children ☐ Parent(s)
tick all that apply

☐ Other ☐
Please specify

4. Please tick one of the housing tenures below that applies to you?

☐ Owned outright ☐ Mortgage / loan ☐ Shared ownership

☐ Rented ☐ Live rent free

☐ Other ☐

Please specify

ID number ☐☐/☐☐☐

5. Which of the following best describes your ethnicity?

White

- ☐ British
- ☐ Irish
- ☐ Gypsy or Irish Traveller
- ☐ Any other white background

Specify ____________

Asian/ Asian British

- ☐ Indian
- ☐ Pakistani
- ☐ Bangladeshi
- ☐ Chinese
- ☐ Any other Asian background

Specify ____________

Other ethnic group

- ☐ Arab
- ☐ Any other ethnic group

Specify ____________

☐ **Prefer not to say**

Mixed

- ☐ White and Black Caribbean
- ☐ White and Black African
- ☐ White and Asian
- ☐ Any other mixed background

Specify ____________

Black or Black British

- ☐ African
- ☐ Caribbean
- ☐ Any other Black background

Specify ____________

ID number ☐☐/☐☐☐

6. Education: Please tick all that apply

☐ 1-4 O levels/ CSEs/ GCSEs any grades NVQ Level1 Foundation GNVQ

☐ 5+ O levels (passes)/ CSEs (grade1)/ GCSEs (grades A*-C) School Certificate, 1A level/ 2-3 AS levels

☐ 2+ A levels/ VCEs, 4+ AS levels, Higher School Certificate

☐ Degree (E.g. BA, BSc)

☐ Higher degree (E.g. MA, PhD, PGCE)

☐ Professional qualifications (E.g. teaching, nursing, accountancy)

☐ NVQ Level 1 Foundation GNVQ

☐ NVQ Level 2, Intermediate GNVQ

☐ NVQ Level 3, Advanced GNVQ, ONC, OND

☐ NVQ Level 4-5, HNC, HND

☐ Apprenticeship

☐ Other qualifications (E.g. City &Guilds, RSA/OCR, BTEC)

Willingness to Change

7. In the past month, have you been actively trying to lose weight?

Yes..... ☐

No...... ☐

8. In the past month, have you been actively trying to keep from gaining weight?

Yes..... ☐

No...... ☐

ID number ☐☐/☐☐☐

9. Are you seriously considering trying to lose weight to reach your goal in the next 6 months?

Yes...... ☐

No....... ☐

10. Have you maintained your desired weight for more than 6 months?

Yes...... ☐

No....... ☐

Physical activity

11. During a typical 7-day period, how many times on average do you do the following kinds of exercise for more than 15 minutes (write on each line the appropriate number).

Times per week

a) Strenuous exercise (heart beats rapidly)
(e.g. Running, jogging, hockey, football, squash, rugby, swimming, long distance cycling) ☐

b) Moderate exercise (not exhausting)
(e.g. fast walking, baseball, tennis, easy cycling, badminton, easy swimming, dancing) ☐

c) Mild exercise (minimum effort)
(e.g. yoga, bowling, easy walking, golf) ☐

12. During a typical 7 day period, in your leisure time, how often do you engage in any regular activity long enough to work up a sweat (heart beats rapidly?)

**Often**	**Sometimes**	**Never/Rarely**
**1.** ☐	**2.** ☐	**3.** ☐

13. Which of the following best describes your daily work activity?

Please tick one box only

I am usually sitting and do not walk about much. ☐

I stand or walk quite a lot but do not have to carry or lift things very often. ☐

I usually lift or carry light loads or have to climb the stairs or hill often. ☐

I do heavy work or carry heavy loads often. ☐

Diet

14. Do you follow a special diet?

Please tick all that apply.

No............... ☐

Yes, because of a medical condition/allergy...... ☐

Yes, to lose weight...... ☐

Yes, because of personal beliefs (religion, vegetarian)...... ☐

Yes, other...... ☐

15. Have you taken any of the following during the past year?

a) Vitamins (e.g. multivitamins, vitamin B, vitamin C, folic acid)

Yes...... ☐

No...... ☐

ID number ☐☐/☐☐☐

b) Minerals (e.g. iron, calcium, zinc, magnesium)

Yes…… ☐

No…… ☐

c) Fish oils (e.g. cod liver oil, omega-3)

Yes…… ☐

No…… ☐

d) Other food supplements (e.g. oil of evening primose, starflower oil, royal jelly, ginseng)

Yes…… ☐

No…… ☐

16. During the course of the last 12 weeks, on average, how many times did you eat the following foods? Please give your answers in the boxes provided.

**Food Type**	**Times/week**	**Examples of a typical portion**
Vegetables (not including potatoes)	☐	medium serving eg 2 broccoli spears, 3 sticks of celery, 3 heaped tablespoons of tinned peas or sweet corn
Salads	☐	medium serving eg 1 desert bowl of mixed salad
Fruit and fruit products (not fruit juice)	☐	medium serving eg 1 apple, 1 pear, 2 satsumas, 1 heaped tablespoon of raisins, currant or 1 handful of dried banana chips
Fish and fish products	☐	medium serving eg 1 salmon, steak, 1 can of tuna, 1 fish from fish & chip shop

Meat, meat products and meat dishes	☐	medium serving eg 1 medium size chicken breast, a lamb chop, 3-4 rashes of bacon, a quarter pound of beef burger

17. In the past 12 weeks have you taken an alcoholic drink:

Please tick one box.

Twice a day or more........................ ☐

Almost daily .... ☐

Once or twice a week........................ ☐

Once or twice a month........................ ☐

Special occasions only..................... ☐

Not at all...................... ☐

18. In a typical 7-day week, including the weekend, how many standard drinks of alcohol do you drink? *(see the table below)*

Please write the number in the box below.

I usually drink _____ standard drinks of alcohol per week

**ONE STANDARD DRINK =**	½ pint of beer
	or ½ pint cider
	or ½ pint lager
	or 1 (125ml) glass of wine, martini, or cinzano
	or 1 small glass of Sherry or Port
	or 1 measure of Spirits (gin, whiskey, vodka etc.)
	or 1 measure liquor
***A PINT OF BEER, CIDER, OR LAGER COUNTS AS TWO STANDARD DRINKS**	
***A DOUBLE MEASURE OF SPIRITS COUNTS AS TWO STANDARD DRINKS**	

19. Do you smoke?

ID number ☐☐/☐☐☐

Yes, I smoke daily………………… ☐

Yes, I smoke occasionally……… ☐

No, I used to smoke……………… ☐

No, I have never smoked……… ☐

20. If yes or you used to smoke, how much, on average, do you (or did you) smoke a day?

Please write in the amount for each; if none write "0"

cigarettes____

cigars____

ounces tobacco____

21. Are you intending to stop smoking in the next six months?

Yes……. ☐

No……. ☐

Lifestyle Information

Please circle the answer you find most fitting to the circumstance.

22. How often do you use the internet in a week?

☐ 1-2 days ☐ 3-4 days ☐ 5-6 days ☐ Everyday

	Yes	No
23. Do you own a smartphone?	☐	☐
24. Do you use a device or app to track physical activity levels?	☐	☐
25. Do you use a device or app for calorie counting and expenditure?	☐	☐

ID number ☐☐/☐☐☐

	Yes	No
26. Do you use a diary for calorie counting and/or energy expenditure?	☐	☐

Previous dieting history

	Yes	No
27. Have you tried dieting in the past?	☐	☐

If yes, what dieting programmes have you tried?
Please tick all that apply

- ☐ BBC healthy living plan
- ☐ NHS choices 12 week weight loss plan
- ☐ Jenny Craig UK
- ☐ Lighter life
- ☐ Sainsbury's Diet
- ☐ Diet chef
- ☐ Dr Atkins diet
- ☐ Slimming world
- ☐ Weight watchers programme
- ☐ Slim-fast plan
- ☐ Tony ferguson weight loss programme
- ☐ Rosemary Conley's eat yourself slim diet and fitness plan
- ☐ Other, please specify ____________

	Yes	No
28. Were you successful in losing some weight?	☐	☐
29. Have you lost 10% or more of your weight in the last 6 months?	☐	☐

Medication history

ID number ☐☐/☐☐☐

30. What medication(s) for diabetes are you currently taking?

Please tick all that apply

- ☐ Metformin
- ☐ Glibenclamide
- ☐ Gliclazide
- ☐ Glimepiride
- ☐ Tolbutamide
- ☐ Acarbose
- ☐ Insulin
- ☐ Diet only,no tablets
- ☐ Other, please specify ______
- ☐ Pioglitazone
- ☐ Vildagliptin
- ☐ Sitagliptin
- ☐ Byetta[R]
- ☐ Victoza[R]
- ☐ Repaglinide
- ☐ Nateglinide

31. Are you taking medications regularly for any condition other than diabetes? Yes ☐ No ☐

If yes, please specify the condition(s)

- ☐ Heart disease
- ☐ High blood pressure
- ☐ High cholesterol
- ☐ COPD or asthma
- ☐ Kidney disease
- ☐ Chronic pain
- ☐ Arthritis
- ☐ Other, please specify ______

Thank you for completing this questionnaire!

ID number ☐☐/☐☐☐

This questionnaire is part of an independent research project within the Collaboration for Leadership in Applied Health Research and Care for South Yorkshire (NIHR CLAHRC SY). The views and opinions expressed are those of the authors, and not necessarily those of the NHS, the NIHR or the Department of Health CLAHRC SY would also like to acknowledge the participation and resources of our partner organisations. Further details can be found at www.clahrc-sy.nihr.ac.uk. © University of Sheffield 2013
