# Supplementary material for: Feasibility study of portable technology for weight loss and HbA1c control in type 2 diabetes
Source: BMC Med Inform Decis Mak. 2016 Jul 15;16:92. doi: 10.1186/s12911-016-0331-2 (PMC4946211; doi:10.1186/s12911-016-0331-2)
Supplement: Additional file 2: — In the file called ‘Additional File 2’ can be found the study’s “Weight loss amongst patients with Type 2 diabetes—Exit Questionnaire” it was an optional questionnaire sent to participants who elected to withdraw from the study. Used to identify reasons for withdrawing. (PDF 238 kb) [file 12911_2016_331_MOESM2_ESM.pdf]

Weight loss amongst patients with Type 2 diabetes

Exit Questionnaire

<table>
<tr><td colspan="2">We are sorry you are leaving the study. We hope you will feel able to complete this exit questionnaire. It is useful to know why you decided to leave the study. It is NOT an attempt to change your mind. Unless you indicate otherwise we will not be in contact with you again after receiving this questionnaire.</td></tr>
<tr><td>Name of Participant:
PLEASE WRITE IN BLOCK CAPITALS</td><td>Signature:</td></tr>
<tr><td>Post Code :</td><td>Date:</td></tr>
</table>

I wish to withdraw my participation in the above named study with immediate effect.

PLEASE TICK ONLY ONE OPTION.

- No further contact: This means that the researchers would no longer contact you about this study, but would still have your permission to retain and use your anonymised data already collected for analyses and in reporting this and future research. ☐

- No future access: This is the same as 'No further contact'above except that you only allow us to use your anonymised data in our current study. ☐

- No further use: This means that the researchers would no longer contact you nor retain or use your data already collected for this research for any purpose. The exceptions ☐

would be :

- your signed consent and withdrawal would be kept as a record of your wishes;
- if you indicated you were willing to participate in other research projects you wish to change that decision too, please let us know in other comments below.
- and finally any published data cannot be recalled or removed.

We would like to improve on the participant experience in further studies and would appreciate it if you would **TICK** all of the following reasons for withdrawal that apply to you. If you have any additional reasons or none of the below apply to you, could you please briefly state the reason in the section titled 'other'.

- There were too many visits to the research facility ☐
- Parking difficulties experienced at the research Facility venue ☐
- Travelling distance to research facility ☐
- I am withdrawing based on advice from my GP ☐
- Due to a medical condition ☐
- I do not feel the device has been of much use ☐
- My expectations when I enrolled in the study have not been met so far ☐
- I have not been allocated a device ☐

- I am intending on using another electronic device or app on my phone to monitor my diet and physical activity ☐

- I did not have enough information about the study before consenting to participate ☐

- I was not given adequate training on the use of the device ☐

- I found the device too difficult to operate ☐

- The device was too burdensome to use ☐

- I did not feel comfortable carrying the device about especially around friends and family ☐

OTHER:

This questionnaire is part of an independent research project within the Collaboration for Leadership in Applied Health Research and Care for South Yorkshire (NIHR CLAHRC SY). The views and opinions expressed are those of the authors, and not necessarily those of the NHS, the NIHR or the Department of Health CLAHRC SY would also like to acknowledge the participation and resources of our partner organisations. Further details can be found at www.clahrc-sy.nihr.ac.uk. © University of Sheffield 2013
